# Buckling assisted and lithographically micropatterned fully flexible sensors for conformal integration applications

**DOI:** 10.1038/srep17776

**Published:** 2015-12-07

**Authors:** Debashis Maji, Debanjan Das, Jyoti Wala, Soumen Das

**Affiliations:** 1 School of Medical Science and Technology, Indian Institute of Technology Kharagpur - 721302, India; 2 Department of Electrical Engineering, Indian Institute of Technology Kharagpur - 721302, India

## Abstract

Development of flexible sensors/electronics over substrates thicker than 100 μm is of immense importance for its practical feasibility. However, unlike over ultrathin films, large bending stress hinders its flexibility. Here we have employed a novel technique of fabricating sensors over a non-planar ridge topology under pre-stretched condition which not only helps in spontaneous generation of large and uniform parallel buckles upon release, but also acts as stress reduction zones thereby preventing Poisson’s ratio induced lateral cracking. Further, we propose a complete lithography compatible process to realize flexible sensors over pre-stretched substrates thicker than 100 μm that are released through dissolution of a water soluble sacrificial layer of polyvinyl alcohol. These buckling assisted flexible sensors demonstrated superior performance along different flexible modalities. Based on the above concept, we also realized a micro thermal flow sensor, conformally wrapped around angiographic catheters to detect flow abnormalities for potential applications in interventional catheterization process.

Advances in flexible electronics demand high degree of stretchability, compressibility and bendability not only of the substrate but also of the overlying thin film sensors/circuits in applications such as robotics, artificial skin, electronic eyes, flexible displays, solar cells, conformal and wearable body sensors, etc^1^,^2^,^3^,^4^,^5^,^6^. Bio-flexible microdevices require the substrates to be biocompatible in addition to being flexible for development of various implantable/semi-implantable devices and sensors^7^,^8^,^9^,^10^,^11^,^12^. In this context, close resemblance of Young’s modulus (~1.0 MPa) of Polydimethylsiloxane (PDMS) elastomer and soft body tissues (~10−500 kPa)^13^ along with its optical transparency, chemical stability and easy pattern generation through replica-molding and soft-lithography facilitate the material as an attractive choice for the intended applications.

Researchers have always tried to exploit the benefit of spontaneous buckling of thin film of inorganic materials when deposited over soft elastomeric substrates like PDMS^14^,^15^. However, low buckling amplitude and wavelength of the as-deposited film have demonstrated low levels of stretchability and tend to generate micro-cracks beyond 1–2% strain leading to total electrical discontinuity beyond 15–20%^15^. In contrast, pre-stretching of elastomeric base has demonstrated extreme stretchability and compressibility in various applications such as diffraction gratings^16^, mechano-responsive optical materials^17^, strain controlled tunable wetting^18^,^19^ and adhesion^20^, stretchable piezoelectric devices^21^, etc. Electronics and sensor applications require thin film metallization over pre-stretched base substrates to achieve superior flexibility as addressed by several groups in the form of selectively adhered Si/GaAs nanoribbons^22^, gold conducting lines^23^, inkjet printed silver lines^24^, etc. However, high amount of pre-stretching leads to lateral cracks over the deposited metal films due to incorporation of Poisson’s ratio induced lateral strain^15^,^23^,^25^ upon release. Although various devices specificity like ultrathin surface, lightweight, transparency or electrically continuous crumpled surface^5^,^26^,^27^ have been successfully addressed in the recent past, realization of stable flexible devices over elastomeric substrates through the simple strategy of utilizing pre-stretch induced buckling of the deposited inorganic thin film have been addressed by very few.

Moreover, among various bio-flexible devices realized till date over PDMS and others, substrate thickness plays an important role in determining sensor flexibility. Good surface conformability with bending radius of curvature <100 μm has been achieved for films of about 1–10 μm thickness^5^,^26^. However, in various applications like flexible microfluidics/liquid-state electronic systems^28^, sensors over microsurgical tools, guide wires^29^, balloon catheters, multi-electrode catheters and ablation catheters for cardiac mapping^9^, etc., processing techniques and final device requirements need thicker substrates (>100 μm) which hinders surface conformality or low bending radius while maintaining stable electrical continuity of the metal films.

Further, patterning over pre-stretched substrates generally involve usage of highly stacked layers, complicated transfer/contact printing^30^ involving wide material knowledge, usage of high end custom build precise equipments^30^,^31^,^32^ and complex process steps^33^ which might limit their further advancement due to various physical and economical factors. Use of shadow mask technique^23^,^34^ remains a popular choice, however, it limits the minimum feature possible to few 100 μm. In comparison, conventional lithography technique is still a preferred solution due to its obvious advantages for bulk processing and achieving submicron feature size.

In accordance with the above needs, our present work highlights a technique for development of large scale, cost effective, highly flexible and stable sensors realized through conventional lithography over a pre-stretched thick PDMS surface capable of conformal bending over highly convoluted surfaces by exploiting the spontaneous buckling of thin film over elastomeric surfaces. We implemented a non-planar ridge topology over the elastomeric surface for micropatterning of sensors over it thereby successfully eliminating lateral cracks over relaxed films. In addition, we demonstrated a stress-free release of individual sensors by dissolution of an underlying sacrificial layer of polyvinyl alcohol (PVA) in water^26^,^35^. High buckle wavelength of ~35 μm and amplitude of ~15 μm of the sensors were achieved by releasing the initial prestrain of 35%. This allowed the sensors to bend down to a minimum radius of ~75 μm around microscopic cover slips maintaining stable electrical continuity even thereafter. Finally, a micro thermal flow sensor was fabricated over a 100 μm thick ridge PDMS elastomer and conformally wrapped around 2 mm diameter angiographic catheters to detect flow abnormalities in a custom built flow setup mimicking that of human artery.

## Results

### Concept

**Figure 1:**
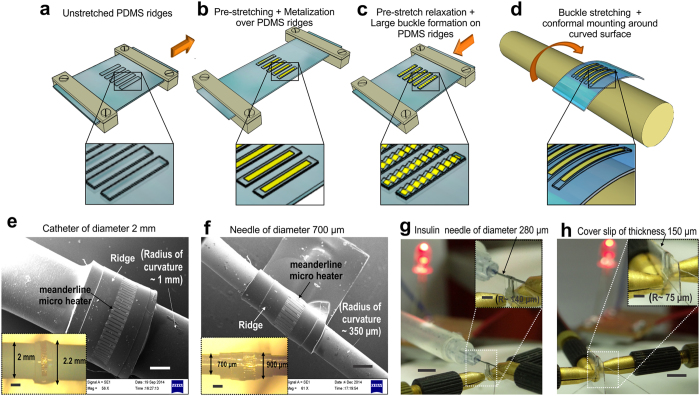
Concept and realization of flexible sensors. Schematic representation of concept of flexible electronics involves **(a)** clamping of a non-planar PDMS ridge topology over a stretching device, **(b)** pre-stretching of the ridge assembly followed by thin film deposition and patterning, **(c)** release of applied prestrain & formation of large parallel buckles and **(d)** flattening up of the buckles upon bending over any curved surface. SEM micrographs of micropatterned resistors over ridges successfully wrapped around **(e)** an angiographic catheter surface (R ~ 1 mm) and **(f)** thin needle surface (R ~ 350 μm). Inset images show the corresponding optical micrographs of the same. 350 μm width thin nichrome microstrips over PDMS ridges, successfully wrapped around (**g)** an insulin needle (R ~ 140 μm) and **(h)** edge of a cover slip of 150 μm thickness (R ~ 75 μm) maintaining stable electrical continuity through the glowing LED bulb thereby demonstrating extreme bendability of the sensors achieved through the above concept. Inset images show the enlarged optical micrographs of the same. Scale bar, 500 μm (**e**,**f**, insets in **e**,**f**), 5 mm (**g**,**h**) and 2 mm (insets in **g**,**h**).

Successful realization of any flexible sensor requires uniform deposition and patterning of thin metal films over soft elastomeric surface maintaining stable electrical continuity when flexed around various directions having small radius of curvature. Figure 1a–d schematically highlights the present concept of realization of flexible sensor. Deposition of thin metal films over elastomeric surface is always associated with the formation of random sinusoidal buckles. Bending of this micro-patterned buckled thin film assembly results in an increase in outer circumferential length as compared to the inner layer, while stretching of the entire assembly also results in an increase in its overall length. This increment in length would cause tensile strain on the surface which, in the present study would be mitigated by stretching up or flattening of the sinusoidal buckles. Hence, magnitude of the buckle wavelength and amplitude must be such that cumulative effect for all the buckles within the length of the sensor should incorporate the increment in the circumferential/lateral length required for complete bending/stretching. However, there are certain prerequisites for achieving sensor flexibility through the above concept.

The primary prerequisite is alignment of the buckles in the direction perpendicular to the bending or stretching i.e. buckle wavelength should be oriented parallel to the stretching direction. This issue may be overcome by generating unidirectional and uniform stress distribution prior to thin film deposition by engineering the top elastomer surface with a non-planar ridge topology. The post deposition stress over this ridge topology^14^ manifests itself preferably along the ridge length and drops across the width leading to unidirectional as well as uniform stress distribution over the ridges. This ensures the formation of sinusoidal buckles parallel to each other and perpendicular to the ridge length. The second prerequisite is controlled buckle amplitude and wavelength required for effective stretching or bending around any curvature. In particular, bending of sensors around very small radius of curvature (<1 mm) like catheter or guide wires demands high deformation of the buckles thereby requiring higher buckle wavelength and amplitude. This may be realized through pre-stretching the PDMS film prior to metallization.

Figure 1a illustrates a non-planar array of ridge topology implemented on the elastomeric substrate for generation of uniform and aligned buckles parallel to each other and to the width of the ridges. The ridges are thereafter subjected to an initial pre-stretch followed by thin film deposition and patterning over the ridge area (Fig. 1b). Gradual release of the initial prestrain resulted in the formation of parallel buckles with large and uniform buckle amplitude and wavelength over the ridge areas (Fig. 1c). These, compressive stress induced large buckles helped to maintain electrical continuity along the entire sensor length over the ridges during stretching, bending or twisting experiments validating superior flexibility of the sensors as shown schematically in Fig. 1d. In the present work, a novel technique of using a sacrificial layer of PVA sandwiched between a 35% pre-stretched film of thickness varying from 100 μm to 700 μm and a rigid secondary substrate was implemented for final release of individual microsensors.

SEM micrographs of Fig. 1e,f demonstrate patterned microheater structure fabricated over a PDMS ridge and wrapped around a catheter and needle surface of diameter 2 mm and 700 μm, respectively. Figure 1g,h illustrates bending of thin straight nichrome strips around 280 μm diameter insulin needle as well as around thin sharp edges of a microscopic cover slip (thickness 150 μm), thereby achieving a radius of curvature down to ~75 μm with stable electrical continuity. The above experiments thus demonstrate extreme flexibility of our sensors, successfully realized through the above technique having numerous potential applications.

### Influence of ridge topology and pre-stretching

Deposition of thin metal films over planar elastomeric surface is always associated with equi-biaxial compressive stress upon cooling due to thermal mismatch between the deposited film and the substrate resulting in formation of random sinusoidal wrinkles or buckles without any preferred direction of orientation^14^ (see Supplementary Data 1). For a 0.245 μm thick nichrome film the buckle wavelength (*λ*_b_) and buckle amplitude (*A*_b_) (peak-to-peak) were measured to be ~10 μm and ~0.9 μm, respectively using AFM scanning of buckles over planar PDMS surface.

**Figure 2:**
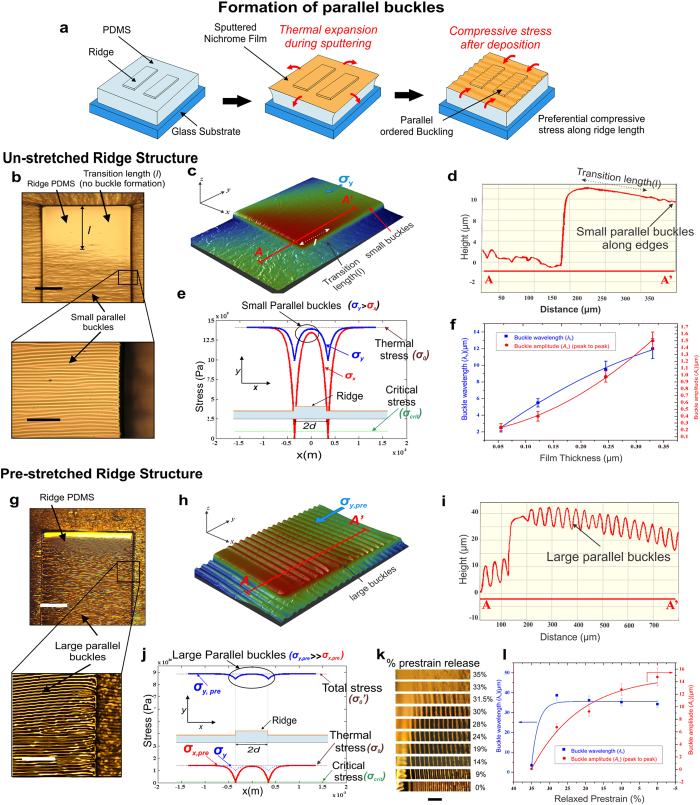
Thin film deposition and stress distribution over un-stretched and pre-stretched PDMS ridge. (**a**) Schematic representation of formation of parallel buckles due to unidirectional stress distribution when thin film is deposited over a non-planar ridge topology. Un-stretched ridge PDMS showing **(b)** development of transition length and formation of small parallel buckles (see magnified image), **(c)** 3D surface profile of a corner of the ridge PDMS showing the initial transition length followed by generation of very fine buckles and **(d)** 2D line profile of the same. **(e)** MATLAB® simulated stress distribution along ridge width (*σ*_x_) and ridge length (*σ*_y_). Only *σ*_x_ (in red) becomes zero near the step walls which results in preferential stress distribution along y-direction (*σ*_y_) (in blue). This increased stress component along y-direction leads to the formation of buckles oriented parallel to each other and to the width of the ridge and **(f)** variation of buckle wavelength and amplitude for different thicknesses of deposited thin film. Pre-stretched ridge PDMS showing **(g)** development of large parallel buckles upon release of the prestrain, **(h)** 3D surface profile of a corner of the pre-stretched ridge PDMS without any transition length followed by generation of large sinusoidal buckles and **(i)** 2D line profile of the same. **(j)** MATLAB® simulated stress distribution for a pre-stretched system shows huge difference in the either stress components with *σ*_y,pre_ ≫ *σ*_x,pre_ due to additional prestrain, resulting in formation of large parallel buckles. **(k)** Sequence of microphotographs of buckled nichrome thin film showing gradual formation of buckles with relaxing prestrain and **(l)** variation of *λ*_b_ and *A*_b_ upon gradual release of the 35% initial prestrain. Scale bar, 250 μm (**b**,**g**), 100 μm (**k**) and 100 μm (magnified images in **b**,**g**).

Unlike planar surface, when steps are present as in ridge topology, the stress distribution in the film remains no longer equi-biaxial but manifests itself preferably along the direction of the ridge length^14^ resulting in the formation of highly ordered buckles parallel to each other and to the width of the ridge as schematically represented in Fig. 2a and shown in the micrograph of Fig. 2b. Figure 2c,d presents the 3D surface scan image and 2D line profile, respectively of a corner of 700 μm width ridge PDMS which prominently shows an initial buckle free length or transition length (*l*) near the edge and thereafter gradual formation of buckles parallel to ridge width, extended along its length. Figure 2e shows a typical plot of the stress distribution in x (*σ*_x_) and y (*σ*_y_) directions over a 700 μm ridge width based on equations (S3 and S4) (see Supplementary Data 2). This shows *σ*_y_ > *σ*_x_ for the entire ridge width with *σ*_x_ becoming zero near the step walls resulting in preferential stress distribution along y-direction leading to the formation of buckles oriented parallel to each other and to the width of the ridge with sinusoidal peaks aligned along the y-axis. *λ*_b_ and *A*_b_ were measured for films of various thicknesses which were found to increase with film thickness as shown in Fig. 2f.

On the other hand, pre-stretching of the ridge substrates followed by thin film metallization results larger *λ*_b_ and *A*_b_, parallel to each other as shown in Fig. 2g due to increased compressive stress upon release. Figure 2h,i shows the corresponding 3D surface image and 2D line scan of the buckles formed after release of the initial 35% pre-stretch resulting in higher *λ*_b_ and *A*_b_ of 35 μm and 15 μm, respectively. Figure 2j shows the simulated stress profile *σ*_x,pre_ and *σ*_y,pre_ over similar ridge structure after complete release of the prestrain obtained through equations (S6 and S7) (see Supplementary Data 2). The results indicate that incorporation of pre-stretching increases *σ*_y,pre_ significantly larger than *σ*_x,pre_, leading to reduction of transition length and formation of large sinusoidal and parallely aligned buckles along ridge width. Moreover, Fig. 2j also includes the simulation plot of *σ*_y_ denoted as original stress in y-direction without any pre-stretching. The result indicates that *σ*_y,pre_ value is much higher than *σ*_y_ or *σ*_x,pre_ due to pre-stretching along y-direction only. It may further be elucidated that simulated *σ*_x,pre_ value is zero at the ridge edge but *σ*_y,pre_ and *σ*_y_ values are nonzero indicating a preferential stress along y-direction only.

Variation of *λ*_b_ and *A*_b_ with decreasing amount of initial pre-strain of 35% to normal unstrained condition was observed in the sequence of microphotographs in Fig. 2k. Figure 2l shows that initial decrease in strain resulted in sharp increase in *λ*_b_ and thereafter remains nearly constant. However, *A*_b_ increases gradually with the release of prestrain level. This result indicates that initial release of prestrain induces a compressive force that squeezes the top layer of metal-PDMS contributing mainly in augmenting the wavelength, while subsequent relaxation contributes predominantly in raising the *A*_b_ than reducing the *λ*_b_. Variation in *λ*_b_ and *A*_b_ were also studied after complete relaxation of different amounts of applied initial prestrain as shown in Supplementary Fig. S1. Increase in initial prestrain from 10% to 35% resulted in reduction of *λ*_b_ from 55 μm to 35 μm and increase in *A*_b_ from 2 μm to 15 μm which indicates that higher prestrain levels contribute significantly in achieving compact buckles with high *A*_b_ and reduced *λ*_b_ and vice versa.

Thus, incorporation of ridge structure satisfied the primary prerequisite of aligned buckles perpendicular to the bending or stretching direction, whereas application of an external prestrain satisfied the second prerequisite of achieving controlled buckle amplitude and wavelength required for effective stretching or bending around small curvatures. It may be noted that metallization over pre-stretched planar films without ridges although produces unidirectional buckles upon release. However, 50% non-uniformity was observed in their amplitude variation in comparison to less than 10% variation over pre-stretched ridge surface (see Supplementary Fig. S2). The higher non-uniformity in the buckle amplitude over pre-stretched planar PDMS may cause cracking over smaller buckles during stretching. Further, such pre-strained planar films were found to be associated with compressive strain induced lateral cracks due to Poisson’s ratio effect which were more predominant for thinner films. On the other hand, incorporation of ridge along with an initial prestrain ensures formation of uniform sinusoidal buckles over the entire ridge length without any lateral cracking.

**Figure 3:**
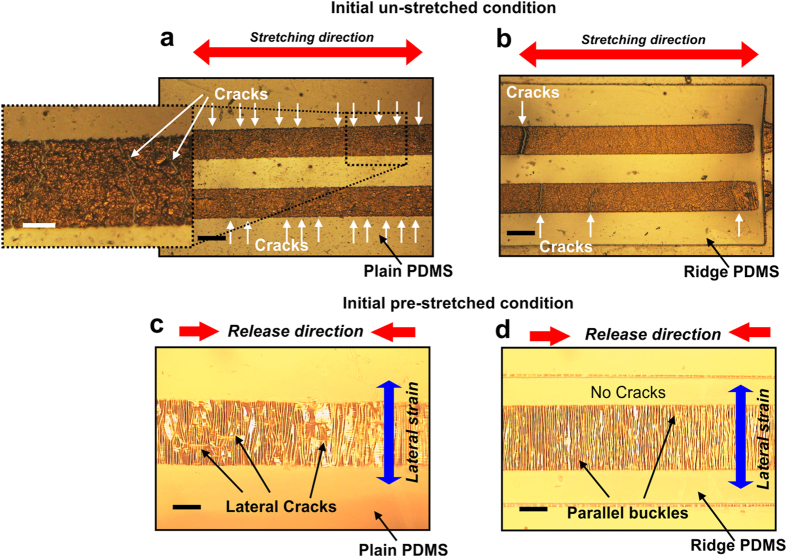
Importance of ridge structure. Microphotographs of 350 μm line width nichrome microstrips fabricated over (**a**) planar PDMS having large number of cracks compared to (**b**) ridge PDMS having few cracks when stretched by 2% along its length from an initially un-stretched condition; Effect of Poisson’s ratio on pre-stretched nichrome microstrips of 350 μm width when released resulting in lateral strain induced (**c**) large crack formation on strips over plain PDMS surface and (**d**) no crack initiation on strips over ridge PDMS surface for 0.245 μm thin deposited films thereby highlighting the importance of ridge. Scale bar, 300 μm (**a**,**b**) and 100 μm (**c**,**d**, magnified image in **a**).

Presence of ridge structure may be considered as small stand-alone elastomeric island over a continuous elastomeric base. During any stretching/bending application, most of the strain is absorbed within the continuous regime, whereas the top islands/ridges experience minimum deformation thereby assisting in preservation of the structural integrity of the sensors developed over the ridges. In order to verify the stress reduction over ridge surface, thin nichrome strips of 350 μm line width and 0.245 μm film thickness were fabricated over the ridges as well as planar PDMS without any initial prestrain and subsequently subjected to a uniform strain using a custom build stretching device. Cracks within the nichrome strips fabricated over planar PDMS were found to initiate at strain levels of about 1% and above 2% strain the number of cracks were almost 3–4 times more than those over the ridges as shown in Fig. 3a,b. Similarly, micro cracks parallel to the nichrome strip length over 35% pre-stretched planar PDMS surface were noticed due to Poisson’s ratio induced lateral compressive strain as compared to crack free strips over pre-stretched ridge surface as shown in Fig. 3c,d, respectively. For thinner film of ~50 nm, numbers of cracks were found to be more over the planar surface while mild crack initiation was observed over ridges. This may be attributed to higher resistance of thicker films to induced lateral strain. The above experiments demonstrate the advantage of incorporation of ridges in not only generating unidirectional and uniform stress induced parallel buckles but also in reducing the stress components during various processes in handling of sensors fabrication.

### Influence of substrate thickness on sensor flexibility

**Figure 4:**
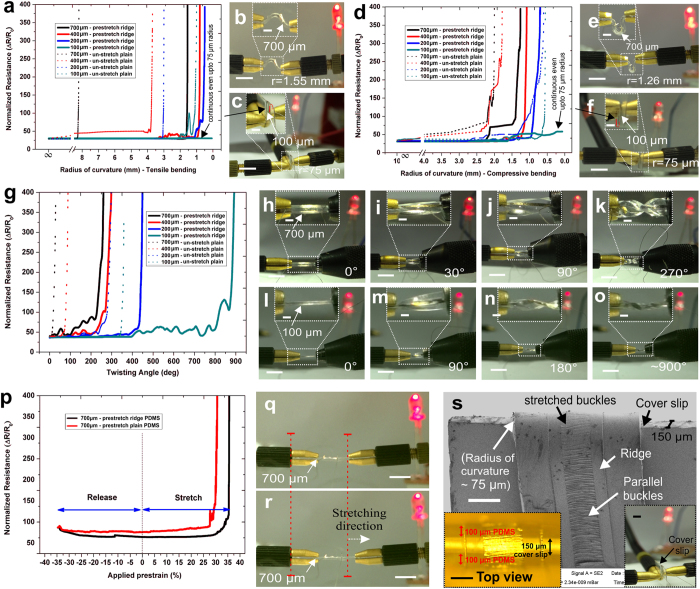
Demonstartion of sensor flexibility. (**a**) Variation of normalized resistance vs. radius of curvature for tensile bending of 350 μm nichrome line width fabricated over un-stretched planar PDMS (dotted lines) surface and pre-stretched ridge (solid lines) surface of 100 μm, 200 μm, 400 μm and 700 μm thickness. Demonstration of electrical continuity through glowing LED upto a minimum bending radius of (**b**) 1.55 mm for 700 μm thick sensor and (**c**) 75 μm for 100 μm thin sensor wrapped around a cover slip. (**d**) Variation of normalized resistance vs. radius of curvature for compressive bending for similar sensors and demonstration of electrical continuity upto a minimum radius of (**e**) 1.26 mm for 700 μm thick sensor and (**f**) 75 μm for 100 μm thin sensor around a cover slip. (**g**) Variation of normalized resistance vs. twisting angle for similar sensors and demonstration of its electrical continuity for 700 μm thick sensor for twisting angle of (**h**) 0°, (**i**) 30°, (**j**) 90° and (**k**) 270° and for 100 μm thin sensor for (**l**) 0°, (**m**) 90°, (**n**) 180° and (**o**) 900° twist. (**p**) Stretchability performance of 350 μm nichrome line width showing variation of average resistance with release of initial prestrain followed by gradual increase of prestrain as demonstrated through the glowing LED in (**q,r**). All measurements of bendability, twistability and stretchability were preformed for minimum of 5 sensors and the data was averaged and normalized thereafter. Fabricated sensors showed high yield of ~90% for sensors over pre-stretched ridges whereas sensors over pre-stretched planar yielded <10% functional sensors due to Poisson’s ratio induced cracks. Sensors over normal ridges as well as planar surface had high yield over thicker substrates than over thinner ones due to improper handling stresses. (**s**) SEM micrograph of 350 μm nichrome strip over pre-stretched ridge bend around a 150 μm thin cover slip edge showing completely flattened buckles (bottom left inset), demonstrating extreme bendability and stretchability with stable electrical continuity (bottom right inset). Scale bar, 5 mm (**b,c,e,f,h-o,q,r**), 1 mm (insets in **b,c,e,f,h-o,** bottom right in **s**), 350 μm (**s**) and 200 μm (bottom left inset in **s**).

Flexibility of any sensor is determined by its bendability (both tensile as well as compressible), twistability and stretchability with stable electrical response. Here, we demonstrated superior flexibility of our sensors along all the different modalities fabricated over PDMS substrates of varying thickness. Thin film sensor/circuits realized over 1 μm thin substrates^26^,^27^ can be easily wrapped conformally around highly convoluted surfaces due to lack of structural rigidity. However, flexibility requirement of several systems needs integration of several layers together making the entire composite assembly thicker than 100 μm for most practical applications. Substrates thicker than 100 μm containing the devices/circuits, generally fail to bend successfully around surfaces with radius of curvature less than 1 mm due to high bending strain over the top surface. As evident from equation (1) (see Methods), thickness of the flexible substrate plays an important role in determining bendability of the film/substrate assembly. To verify the above observation experimentally, nichrome microstrips of 350 μm line width were fabricated over 35% pre-stretched ridges as well as on planar PDMS surface (without pre-stretch) of substrate thickness varying from 100 ± 5 μm to 700 ± 20 μm. Individual sensors were mounted over probe holder arms and electrical resistance of the nichrome microstrips were recorded by reducing the radius of curvature. The sensors were gradually subjected to both tensile loading i.e. with sensors present over the outer surface (Fig. 4a–c) and compressive loading i.e. with sensors present over the inner surface (Fig. 4d–f) by approaching the probe arms nearer to each other at a uniform rate of about 1 mm/min. Figure 4a,d shows the variation of normalized resistance with bending radius of curvature under tensile and compressive loading over the sensors, respectively for various stretched and un-stretched sensors. It was observed that for un-stretched sensors, minimum bending radii of 1 mm and 8.2 mm were obtained for 100 μm and 700 μm thick sensors, respectively under tensile loading. These results match well with the corresponding theoretical results of 1.1 mm and 7.8 mm, respectively computed through equation (1). Similarly, compressive loading resulted in a minimum radius of 0.55 mm and 2 mm, respectively. On the other hand, pre-stretched sensors of 700 μm thickness showed a minimum bending radius of 1.55 mm and 1.26 mm for tensile and compressive loading, respectively whereas 100 μm thick pre-stretched sensors could successfully bend upto 75 μm radius around the edge of a 150 μm thick microscopic cover slip for both tensile and compressive loading maintaining good electrical continuity even thereafter.

Further, similar samples were subjected to gradual twisting with one probe arm fixed and the other rotating at about 30 degree/min. The variation of normalized resistance against the twisting angle is shown in Fig. 4g. 700 μm thick pre-stretched sensors were found to be electrically continuous upto a maximum twist of 270 ± 10 degrees (Fig. 4h–k) whereas, 100 μm sensors exhibited capability to sustain enormous twist of >900 degree (Fig. 4l–o). It was observed further that 100 μm thin sensors got practically crumbled upon heavy twisting (>900) but were still electrically continuous as indicated through the LED lighting on the background.

While testing, linear stretchability, it was observed for all the cases that the maximum stretchability sustaining proper electrical continuity was upto the initial prestrain level i.e. 35% in this case. Figure 4p–r shows variation of average resistance of a 700 μm thick sensor as the applied prestrain was uniformly decreased from an initial prestrain of 35% to 0% followed by gradual increase beyond 35% at a uniform rate of about 4%/min. From the figure it may be observed that the resistance decreases initially with release of the applied prestrain and thereafter remains almost constant for most of the prestrain region. Thin microstrips over pre-stretched ridges, showed very little variation in resistance change with increasing prestrain till the initial applied prestrain of 35% and thereafter increased significantly beyond 37%. In comparison, the thin strips over pre-stretched plain PDMS showed larger variation upon stretching and became electrically discontinuous within 30% prestrain. Un-stretched sensors showed electrical discontinuity within 2% stretching as observed in Fig. 3a,b. The higher stretchability of the structures over pre-stretched ridges may be attributed to the reduction in the Poisson’s ratio induced lateral strain over ridges thereby delaying the initiation of crack formation as compared to that over planar PDMS surface. Figure 4s shows the SEM micrograph of 350 μm width nichrome microstrip over a 100 μm thick PDMS bend around the edge of a microscopic cover slip confirming presence of long parallel buckles at the lower ends of the nichrome strips which gradually flattened/stretched near the edge of the cover slip without formation of cracks and showed stable electrical continuity (bottom right inset of Fig. 4s). Our sensors thus, demonstrated successfully high stretchability around sharp edges as well as extreme bendability of 75 μm radius highlighting superior flexibility of the devices.

### Quantitative estimation of sensor flexibility through pre-stretching

**Figure 5:**
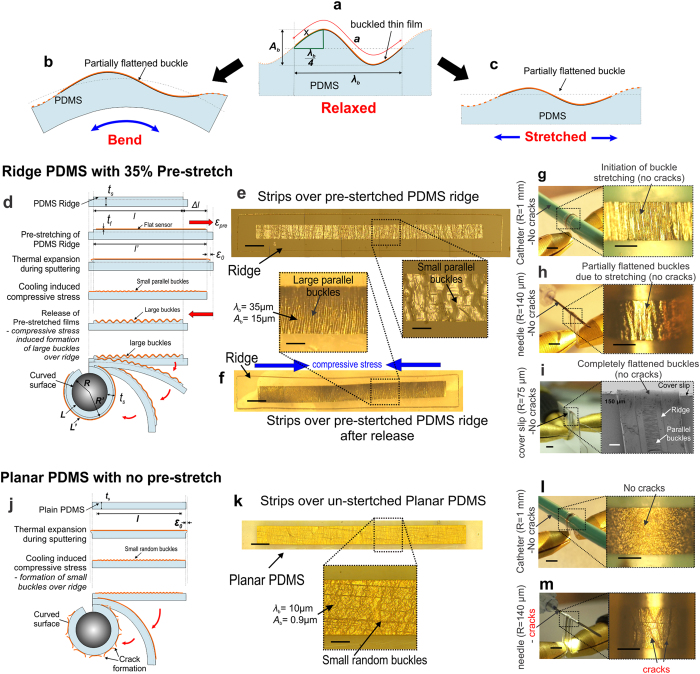
Quantitative modeling of individual buckle and estimation of sensor bendability. (**a**) Schematic representation of an individual buckle showing buckle wavelength (*λ*_b_), amplitude (*A*_b_) and sinusoidal buckle length ‘a’ governing the maximum stretchability of the buckled sensors with flattening up of the buckles upon (**b**) bending and/or (**c**) stretching. (**d**) Schematic illustration of extreme bending of pre-stretched ridge PDMS resulting in gradual flattening-up of the buckles without any cracking of the top film upon higher bending. Microphotographs of fabricated nichrome microstrips (**e**) over pre-stretched ridge with magnified image showing formation of small parallel buckles and (**f**) upon release of the prestrain with magnified image showing formation of large parallel buckles (*λ*_b_ = 35 μm and *A*_b_ = 15 μm). Successful bending of fabricated microstrips over various surfaces like (**g**) catheter (radius of 1 mm), (**h**) insulin needle (radius of 140 μm) and (**i**) edge of cover slip (thickness of 150 μm) with decreasing bending radius of curvature. Magnified optical microphotographs of (**g**,**h**) and SEM image of **i** shows no cracks formation with initiation to complete flattening of buckles. (**j**) Schematic illustration of low levels of bending without any pre-stretching over planar PDMS resulting in cracking of the top film upon higher bending. (**k**) Microphotographs of fabricated microstrips over un-stretched ridge PDMS with magnified image showing formation of small random buckles due to thermal stress. Bending of microstrip over (**l**) catheter (radius 1 mm) surface showing no formation of cracks and over (**m**) insulin needle (radius 140 μm) with severe crack formation after bending. Scale bar, 300 μm (**e**,**f**,**k**), 100 μm (magnified images in **e**,**f**,**k**), 2mm (**g**,**h,i,l,m)** and 200 μm (magnified images in **g**,**h,i,l,m**).

Theoretical estimation of tensile bending of un-stretched sensors, over 100 μm thick PDMS surface resulted in a minimum bending radius of 1.1 mm using equation (1). However, for pre-stretched systems extreme bendability for 100 μm thick sensor upto ~75 μm radius may be successfully addressed through mathematical modeling of individual buckles (see methods section) as represented schematically through Fig. 5a–c. As calculated theoretically, through equations (2, 3, 4, 5, 6) (see methods section), the minimum tensile bending radius, *R* can be estimated to be ~230 μm. However, experimental observation showed that the buckles did not completely flatten up for *R* ~ 230 μm which may be due to compressive stress induced creep in the metal-elastomer interface. This allowed for further bending, yielding a much lower tensile bending radius of not only upto 140 μm around an insulin needle where the buckles gradually started flattening up (Fig. 5h) but even extreme bendability of upto 75 μm radius around the edge of a 150 μm thick microscopic cover slip where the buckles were observed to completely flatten up (Fig. 5i). In contrast, plain PDMS without any pre-strain, results in the formation of small buckles (Fig. 5j,k) after cooling subsequent to deposition process, which could sustain only larger bending radius around catheter (Fig. 5l) and developed cracks when bend to smaller radius like an insulin needle (Fig. 5m) and beyond.

### Preparation of lithography compatible pre-stretched substrates and sensor micropatterning

**Figure 6:**
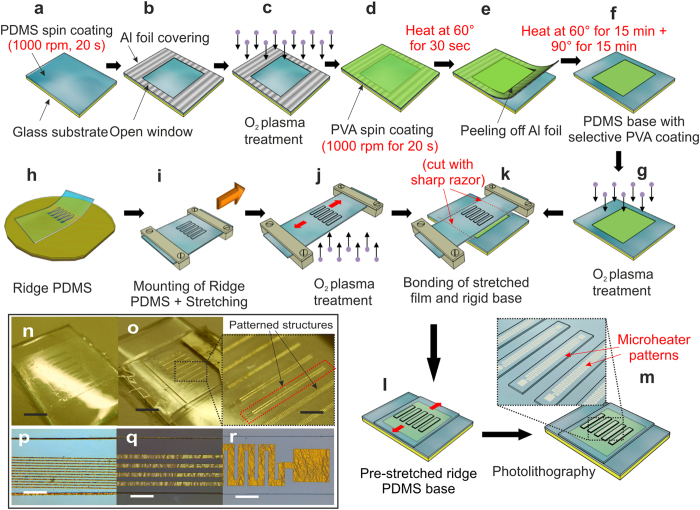
Preparation of lithography compatible pre-stretched substrates for realization of flexible sensors. Schematic representation of preparation of rigid secondary substrate involving (**a**) PDMS spin coating over pre cleaned glass substrates, (**b**) covering with Al foil having a square opening over the PDMS film followed by (**c**) surface activation of the exposed region using oxygen plasma and (**d**) spin coating of the sacrificial PVA layer. (**e**) Gradual peeling-off of the Al foil followed by (**f**) further heating resulting in (**g**) formation of rigid PVA Island of ~2 μm thickness over PDMS base/substrate used for micropatterning process. (**h**) Gradual peeling-off of PDMS ridge structures from SU8 5 master mold. (**i**) Mounting and (**j**) stretching of a PDMS ridge film over a stretching device. (**k**) Conformal fixation of the stretched film over the rigid secondary base, (**l**) final pre-stretched assembly ready for the (**m**) subsequent photolithography process and pattern generation. Optical micrographs of (**n**) pre-stretched ridges over rigid secondary base prior to lithography and (**o**) patterned micro-structures over pre-stretched surface after lithography resulting in generation of various features like line strips as small as (**p**) 10 μm width and (**q**) 50 μm width and (**r**) meanderline resistors fabricated over ridge structures. Scale bar, 5 mm (**n**,**o**), 2 mm (magnified image in **o**) and 250 μm (**p**,**q**,**r**).

Realization of flexible sensor over a pre-stretched substrate requires a rigid secondary base for subsequent micropatterning through photolithography as schematically represented in Fig. 6a–m. PDMS films of about 120 μm thickness were spin-coated and polymerized over pre-cleaned glass substrates (Fig. 6a) (see methods) followed by covering with an aluminum (Al) foil having a square window at the centre for surface modification using oxygen plasma^36^ (Fig. 6b,c). Subsequently, a water soluble sacrificial layer of PVA was spin coated and heated by two step process to get a hardened PVA island of ~2 μm thickness over the base PDMS forming a rigid secondary substrate as shown schematically in Fig. 6d–g. Parallely, a non-planar ridge topography having rectangular ridges of 15 μm height over 85 μm thick base PDMS surface was realized using soft-lithography replica molding process (see Methods section). A thick layer of PDMS containing ridge structures generated from SU8 master template (Fig. 6h) were attached to the two ends of a custom build stretching device with the ridge side facing top as shown in Fig. 6i. Keeping one end of the device fixed, the other end was stretched uniformly using a rotating screw at a uniform rate of about 0.5%/s to achieve a final strain of about 35% (Fig. 6j). Subsequently, the back surface of pre-stretched PDMS ridge substrate (Fig. 6j) as well as the top surface of the rigid secondary substrate containing PVA island (Fig. 6g) were treated with oxygen plasma and the two activated surfaces were placed in close proximity followed by proper alignment to position the PVA island beneath the array of stretched ridges and immediately brought into conformal contact (Fig. 6k). The entire assembly was kept under a mild uniform pressure from top and bottom and gradually released from the stretching device followed by heating at 90 °C in a hot plate for 1 hr to enhance bonding of the two surfaces. In this way a mechanically robust PDMS ridge surface under stretched condition was successfully fixed over a rigid secondary glass substrate as shown in Fig. 6l making it a stand-alone assembly compatible for subsequent metallization and micro-patterning through photolithography process as shown in Fig. 6m. Figure 6n shows the image of a pre-stretched ridge substrate prior to deposition process. Figure 6o–r shows various fabricated structures of varying microstrip line widths and geometries over arrays of pre-stretched ridges with a minimum feature size of 10 μm nichrome strips.

The embedded PVA island at the centre acted as a sacrificial layer for the top stretched ridges, while the PDMS edges surrounding the PVA island facilitated bonding with the top stretched film withstanding the prestrain and keeping the ridge structures under stretched condition even after removal from the stretching device. Thickness of the base PDMS may assume to play an important role in assuring robust bonding of the top pre-stretched film with it. It was observed that for thicker pre-stretched substrates, the composite assembly delaminated from the glass support due to increased compressive stress (see supplementary Fig. S3a,b). The above issue was resolved by peeling off the base PDMS layer after spin coating and polymerization, followed by plasma bonding with the glass substrate again. This ensured better adhesion and made the final composite assembly having varying thickness of pre-stretched film independent of the base PDMS thickness (see supplementary Fig. S3c,d).

### Feasibility study of a micro thermal flow sensor over pre-stretched substrate

**Figure 7:**
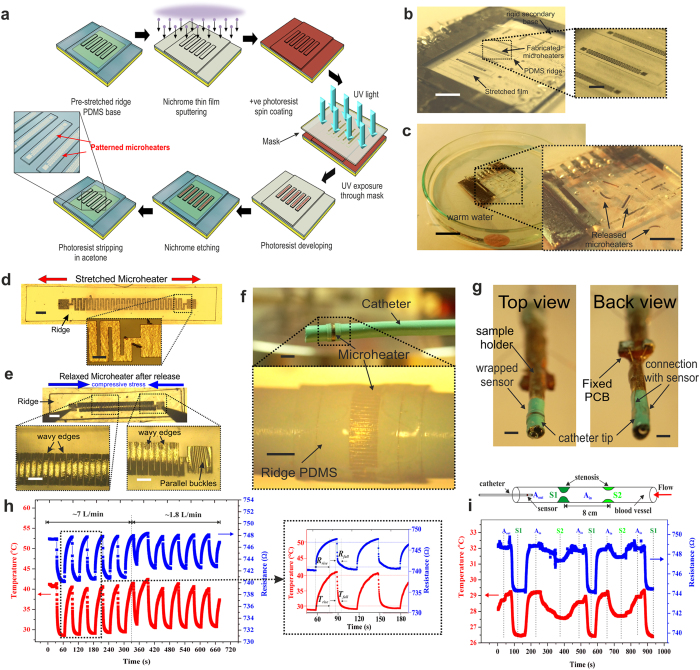
Demonsration of flexible flow sensor mounted over an angiographic catheter. (**a**) Fabrication procedure for a micro thermal flow sensor developed over pre-stretched substrates using conventional lithography involving, nichrome sputtering, photoresist spinning, UV exposure, developing and etching. (**b**) Microphotograph of arrays of fabricated microheaters over pre-stretched ridge and magnified image of the same. (**c**) Release of individual sensors under warm water with magnified view of the released sensors. Microphotographs of fabricated microheater (**d**) over pre-stretched ridge PDMS and (**e**) over pre-stretched ridge PDMS upon release of the initial prestrain. Magnified images shows formation of small parallel buckles in (**d**) and large parallel buckles in (**e**). (**f**) Mounted sensor over the catheter tip with magnified view of the meanderline microheater pattern. (**g**) Portion of the catheter tip containing the sensor mounted on a sample holder showing top and back view of the electrical connections from the sensor terminals to the external signal conditioning circuit through an intermediate PCB board. A J- Type thermocouple was mounted at close proximity to the sensor surface to measure the temperature change associated with the flow. Variation of resistance and temperature profile of the microheaters (**h**) for low and high pulsatile flow rate of 7 L/min and 1.8 L/min, respectively showing greater fall in sensor resistance and temperature with increase in flow rate and vice versa. The magnified graph in (**h**) shows the variation of sensor resistance and temperature with pulsatile flow indicating a sensor response time (R_rise_) of 20 s and (R_fall_) of 8 s. (**i**) Variation of sensor resistance and temperature at a flow rate of 1.8 L/min as the sensor/catheter assembly is gradually moved between the normal regions and different stenosis regions, S1 (50%) and S2 (25%) as schematically marked in the tube. Higher variation near larger stenotic regions is observed as compared to lower stenotic or normal regions. Scale bar, 5 mm (**b**), 1 mm (magnified image in **b**), 2 cm (**c**), 5 mm (magnified image in **c**), 400 μm (**d**,**e**), 100 μm (magnified images in **d**,**e**), 2 mm (**f**,**g**) and 500 μm (magnified image in **f**).

In this section, we present realization of a flexible flow sensor over a 100 μm thick and 35% pre-stretched ridge PDMS elastomer by conventional photolithography technique followed by conformal integration around a 2 mm diameter angiographic catheter for fluid flow measurement. Pre-stretched ridge substrate was used for realization of micro thermal flow sensors as schematically outlined in Fig. 7a. Nichrome thin film was sputtered under optimized conditions over oxygen plasma treated pre-stretched PDMS ridge assembly to achieve crack-free continuous thin films^37^. The stretched ridge assembly was mechanically very stable and showed no stress induced mechanical deformations even after being exposed to the sputtering conditions. Standard positive UV photolithography was employed to pattern the sensors (see Methods section for details). Precaution was taken to carefully align the resistor patterns over the stretched ridges using Mask 2 (see Methods section). Following realization of microheater patterning (Fig. 7b) over individual ridges, the PDMS located between two ridges were cut and removed (Supplementary Fig. S4). Subsequently the whole assembly was dipped under warm water at 60 °C with occasional stirring for ~1hr to facilitate easy release of the sensors as shown in Fig. 7c. The pre-stressed sensors curled-up upon dissolution of the PVA layer due to gradual release of the prestrain and spontaneously generated large parallel buckles over the sensor surface (see Supplementary Video) as compared to that under unstretched condition (Fig. 7d,e). Individual polymer chips were fished out from water and dried under a warm ambient condition. Partially polymerized PDMS mixture was used as a glue to fix individual sensors over cardiovascular angiographic catheters (obtained from Pro-Flo^®^ 6F JR 3.5, Medtronic) followed by moderate heating to complete the polymerization and bond the sensor over the catheter surface (Fig. 7f) (for details see Methods section). The measured resistance was 750 ± 10 Ω for different resistors over the same sensor array. A small portion of the catheter tip containing the mounted sensor was cut and attached to the tip of a customized sample holder for flow measurement as shown in Fig. 7g. Stable electrical connections were ensured to record the sensor output voltage, current (see methods section) and thermocouple temperature in real time using a high speed data acquisition unit.

Variation in the pulsatile flow measurements was conducted at different air velocities. Figure 7h shows the variation in output resistance and temperature for pulsating flow rates at ~7 L/min and ~1.8 L/min. A higher drop in resistance and temperature of ~8 Ω and ~12 °C, respectively was observed for high flow rate as compared to ~6 Ω and ~ 9 °C, respectively for low flow rate.

Detection of degree of stenosis for a low flow rate of 1.8 L/min was also performed. A custom made transparent hollow pipe with internal diameter of 2 cm having two concentric ring shaped obstructions with opening of diameter 1 cm (S1) and 1.5 cm (S2), mimicking the formation of 50% and 25% stenosis was built for detection of flow anomalies and feasibility study of the flow sensor as shown in the schematic of Fig. 7i. One end of the flow tube was connected to a compressor unit through a pressure gauge to ensure controlled flow of compressed dry air (CDA) while the other end was kept open for insertion of the sensor/catheter assembly. Figure 7i shows the variation of resistance and temperature of the microheater as the sensor/catheter assembly was inserted gradually from the outer normal region (A_out_) to 50% stenosis area (S1) and further to inner normal region (A_in_) and finally 25% stenosis region (S2). It was observed that S1 stenosis showed greater drop in resistance of ~5 Ω and temperature drop of ~3 °C than for S2 which showed a drop in ~2.5 Ω resistance and ~1.5 °C of temperature.

Thus, significant detection of the above flow variation in obstructed as well as normal region for both low and high flow rates clearly demonstrates the potential application of the above flexible flow sensor in detecting abnormalities in a blood vessel for successful usage in biomedical healthcare applications.

## Discussion

In the present study, we have demonstrated a cost-effective photolithography compatible method for realization of bio-flexible devices successfully addressing various issues relevant to its effective commercialization. We achieved successful micropatterning by conventional lithography technique upto feature size as small as 10 μm over thick (100 μm to 700 μm) pre-stretched non-planar PDMS elastomeric surface and effectively utilized thin film buckling phenomenon to provide sensor flexibility. In addition, we employed a non-planar ridge topology over the elastomer surface to generate highly ordered buckles and to reduce stress effects over the fabricated microstructures. Present strategy not only helped to overcome a major problem associated with release of pre-stretched films i.e. elimination of lateral cracks due to Poisson’s ratio effect, but also may assist in designing flexible film subjected to even higher prestrain levels upto 100% and beyond. Further, a process for easy and stress-free release of the pre-stretched sensors after its complete realization using a water soluble sacrificial layer of PVA was implemented.

Utilizing these novel strategies, we successfully demonstrated extreme flexibility of our sensors with high twistability of >900 degrees and bendability upto 75 μm radius, beyond their theoretical limit and showed a proof-of-concept of its application through the development of a functional miniature flexible flow sensor conformally wrapped around a 2 mm diameter catheter tip and 150 μm diameter insulin syringe needle for detection of flow abnormalities. Thus, these novel techniques along with use of direct lithography to generate sub-micron patterns over pre-stretched substrates may be further explored in various applications other than flexible electronics like microfluidics, optics, bio-mechanics, robotic surgery, structural mechanics in dynamic equilibrium, etc., thereby effectively merging new innovative techniques with conventional standard methods for widespread technological outreach.

## Methods

### Mask designing for flow sensor

Realization of micro flow sensor over pre-stretched ridges involved a two mask process. Mask 1 consisted of arrays of rectangular ridges of width 700 μm and length 6.14 mm in order to wrap entirely around a 2 mm diameter catheter. Mask 2 involved arrays of meanderline microheater pattern which was designed to align with the 35% pre-stretched PDMS ridge arrays. Considering an external prestrain of 35% and Poisson’s ratio of PDMS to be 0.48, Mask 2 was designed with individual resistor lengths being 35% larger than under un-stretched condition, along with concomitant reduction along the resistor array width to account for the lateral contraction due to Poisson’s effect.

### Preparation of rigid secondary substrate

Glass substrates obtained from HIMEDIA (CG007-0) were used as the rigid secondary substrates for fixing the pre-stretched elastomeric film as well as providing support for the subsequent lithography process. Glass substrates were thoroughly cleaned with piranha solution (H_2_O_2_:H_2_SO_4_−1:1) for 15 min followed by rinsing in DI water thoroughly and heating in convection oven at 150 °C for 30 min. PDMS mix in 10:1 ratio of base and curing agent was spin coated over the pre-cleaned glass slides at 1000 rpm for 20 s and cured at 120 °C for 2 hr in a convection oven to ensure complete polymerization. Thickness of the resulting PDMS secondary substrates was about 120 ± 5 μm. A sacrificial layer of 10% PVA (hot water soluble from HIMEDIA) was spin coated over the activated PDMS window at 1000 rpm for 20 s and heated to get a hardened PVA island of ~2 μm thickness above the PDMS surface.

### SU8 master template preparation

SU8 5 photoresist from Microchem was spin coated over pre-cleaned Si substrates at 500 rpm for 10 s followed by 1000 rpm for 20 s. Samples were then prebaked at 65 °C for 2 min followed by softbaking at 95 °C for 5 min. Subsequently the samples were exposed to UV light (Karl Suss MA6 Mask Aligner) through the chrome mask (Mask 1) containing the ridge patterns for 12 s. Post exposure baking was done at 65 °C for 1 min and at 95 °C for 2 min, followed by developing using a developer solution for 40 s and rinsing in IPA (iso-propyl alcohol) for 30 s. The resulting SU8 pattern contained rectangular wells of depth 15 ± 2μm as measured using a surface profiler (Dektak 150 Surface Profiler, Veeco Instruments, Inc.) and were used as master template to obtain PDMS ridges through soft lithography.

### Non-planar topology over PDMS

PDMS mix (Sylgard 184, Dow-Corning Corporation, USA) consisting of prepolymer base and curing agent in 10:1 ratio by weight was mixed, degassed in vacuum for 15 min and spin coated at 1200 rpm for 20 s (Laurell technologies, WS-650) over previously fabricated SU8 rectangular wells of 15 ± 2 μm depth over silicon substrates. PDMS ridge height was optimized to ensure uniform spreading of photoresist over and around the ridges during the photolithography process. PDMS mould was peeled off from the master after complete polymerization at 120 °C for 2 hr in a convection oven. The SU8 wells thus reproduced themselves as PDMS ridges having ridge height ~15 μm over a base PDMS layer of ~85 μm thicknesses. Thus the total thickness of the sensor substrate was 100 ± 5 μm as measured by a surface profiler.

### Optimization of Non-planar PDMS topology

Optimization in ridge length was chosen as per the wrapping length around the catheter of diameter 2 mm which in the present application of a flow sensor was 6.14 mm for a circumferential length of 6.28 mm. Width was chosen as 700 μm, as per the design requirements for proper accommodation of the sensor, ensuring formation of uniform and parallel buckles after release from initial pre-stretch. Wider ridges resulted in non-uniform buckles similar to supplementary Fig. S2a,c and also showed lesser effect in reducing Poisson’s ratio induced lateral crack, whereas narrower ridges resulted in greater chances of misalignment of the pre-stretched ridges with the subsequent patterning mask. Finally, ridge height was optimized to within 15–30 μm to ensure uniform spreading of photoresist over and around the ridges during the photolithography process and 15 μm was chosen in the present case. Higher ridge height hindered the uniform spreading of photoresist during spinning resulting in no resist over the ridges areas, particularly over the ridges away from the centre. In addition, higher ridges of ~100 μm resulted in non-uniform coating of resist when coated with greater volume of resist and spun at low spin speed with the edges of ridge forming a pool of excess resist. Subsequently, after development the resist not only covered the patterned areas but also remained as a pool of liquid on either edge. This led to un-etched metal parts not only along the patterns but also at the edges over the ridge as shown in the supplementary Fig. S5. On the other hand, smaller ridge height acted almost as planar surface and provided very little stress discontinuity particularly for higher film thickness resulting non-uniform buckle generation.

### Surface modification of PDMS

PDMS surface being highly hydrophobic was treated with oxygen plasma in FEMTO system from Diener Electronics for 30 s at RF power of 40 W to increase the surface energy and render the surface hydrophilic prior to PVA coating or thin film deposition through sputtering.

### Nichrome deposition

A 4 inch diameter nichrome sputtering target of composition Ni:Cr 80:20 and purity 99.998% was used as the cathode to deposit nichrome. Nichrome thin film was sputter deposited over plasma treated pre-stretched PDMS ridge substrate assembly under optimized conditions of 60 W power and 0.009 mbar sputtering pressure for 10 min to achieve crack-free continuous thin films of ~0.25 μm thickness.

### Photolithography over stretched substrate

Positive photoresist, HPR 504 from Fujifilm was spin coated over the nichrome deposited pre-stretched PDMS ridge surface at 3000 rpm for 20 s followed by prebaking at 90 °C for 30 min. Care was taken to ensure conformal coverage of the photoresist over the ridge as well as the base. Samples were then exposed to UV for 7 s (Karl Suss MA6 Mask Aligner) through Mask 2 containing the microheater patterns. Subsequently the resist was developed in HPRD 429 to achieve patterned structures followed by postbaking at 110 °C for 30 min instead of recommended 120 °C for positive resist process since the higher temperature often lead to development of cracks over the photoresist surface. Etching was performed by nichrome etchant consisting of H_2_O: H_2_O_2_: HNO_3_: HCl in 1:1:2:2 ratio with an etch rate of about 0.5 μm/min. The etched samples were often found to be associated with a very thin rainbow colored film of chromium which was removed by dipping the samples in chromium etchant for about 5 min. Finally, the positive photoresist was stripped off using warm acetone exposing the fabricated nichrome microheaters over transparent PDMS surface.

### Individual sensor release

In order to facilitate easy seepage of water under the ridge area, the stretched PDMS between the ridges (containing microheaters) as well as surrounding regions were cut off using a sharp razor and manually peeled off. Thereafter, the ridges containing the sensors were dipped under warm water at 60 °C with occasional stirring for ~1hr to enable complete dissolution of the underlying PVA layer and facilitate stress-free release of individual sensors over PDMS ridge islands.

### Measurement of buckle wavelength and amplitude

Buckle wavelength (*λ*_b_) and amplitude (*A*_b_) was measured for an unstrained ridge/planar PDMS surface using an AFM (MultiMode 8 Scanner with NanoScope V Controlller). Scanning was performed in tapping mode using RTESP - 300 tip (Bruker AFM Probes) having a force constant of 40 N/m at a resonance frequency of 300 kHz. Images were scanned at 256 × 256 pixels for a scan area of 50 μm × 50 μm over the PDMS surface. For an initial pre-stretched ridge PDMS, large buckle wavelength and amplitude formed after release was scanned for both 2D line profile as well as 3D surface profile using a surface profiler (Dektak 150 Surface Profiler, Veeco Instruments, Inc.) with stylus tip of diameter 2.5 μm and applied force of 0.5 mg having a scan resolution of 0.056 μm/sample.

### Sensor integration over catheter

Electrically continuous microheater structures over ridges were used for wrapping around the catheter tip for realization of a flexible flow sensor. Partially polymerized PDMS mixture was used as a glue to fix individual sensors over the catheter surface. Cardiovascular angiographic catheters was mounted horizontally under the probe station using a tungsten carbide probe of 25 μm tip diameter acting as a support and then applying a small drop of PDMS mixture over the catheter surface. Subsequently the polymer chip was brought near it using a tweezer and one end was carefully placed over the mix and held in position using two additional probes acting as mounting probes as shown in supplementary Fig. S6a. The assembly was allowed to heat under a halogen lamp for 30 min placed at a distal location to completely polymerize the section of the glue and fix the sensor end firmly over the catheter. After releasing the mounting probes, the catheter with sensor was slightly rotated and the PDMS mix was applied again at a new location along the wrapping diameter. Subsequently, the sensor was gradually bent using the probes and held fixed over the applied glue area followed by exposure to the halogen lamp for polymerization and fixation. This process was repeated for about four times till the other end of the ridge was fixed over the catheter surface as shown in supplementary Fig. S6b. After complete mounting, the entire assembly was observed carefully for any crack formation and to confirm for electrical continuity.

### Stable electrical connections

Electrical connections were obtained from the sensors by connecting the bond pads to 1 mill copper wires using silver conductive adhesive paste (from Alfa Aesar - R_s_: <0.025 Ω/□ @ 0.001 inch thick) and soldering the other end to an intermediate small printed circuit board (PCB) (5 mm × 5 mm) before taking out the final wiring to the external electronics comprising of a potential divider circuit. Thermocouple temperature and output voltage and current from the flow sensor were measured in real time using a high speed data acquisition unit (Agilent 34972A).

### Determination of minimum radius of curvature

For a composite assembly like hard metal thin film over soft elastomeric substrate having different material thickness and Young’s modulus such that *t*_f_ < *t*_s_, and *E*_f_ > *E*_s_, where *t*_f_, *t*_s_, *E*_f_ and *E*_s_ are the thickness and Young’s modulus of the film and substrate respectively, the radius of curvature upto which the composite can be bend without causing any fracture to the top film is given by the modified Stoney’s formula^38^ represented by


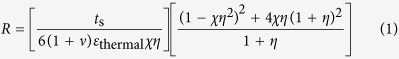


where thermal strain, *ε*_thermal_ = (*α*_f_ – *α*_s_) Δ*T* and *α*_f_ and *α*_s_ are the thermal coefficient of expansion of the deposited film and the substrate respectively and *η* = *t*_f_/*t*_s_ and *χ* = *E*_f_/*E*_s_. Minimum radius of curvature were computed considering other parameters to be *E*_f_  = 213 GPa, *E*_s_ = 1.87 MPa (for bulk PDMS)^37^, *t*_f_ = 0.245 μm, ν = 0.3 for the film, *α*_f_ = 15 × 10^−6^ °C^−1^ and *α*_s_ = 310 × 10^−6^ °C^−1^ and considering sputtering at ~180 °C.

### Mathematical modeling of an individual buckle

Figure 5a schematically illustrates a typical sinusoidal buckle of wavelength, *λ*_b_ and amplitude, *A*_b_ (peak to peak). Let ‘a’ be the total length of the sinusoidal path along the buckle which is also equal to the maximum length that a single buckle can be flattened during bending or stretching (Fig. 5b,c). Therefore increment of a single buckle Δ*a* by stretching along its length is given by


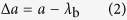


where *a* and *x* is given by,


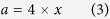


and


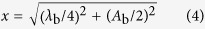


obtained by approximating each quarter wave as a right angled triangle as shown in Fig. 5a. For a 35% pre-stretched PDMS ridge (Fig. 5e), *λ*_b_ and *A*_b_ are 35 μm and 15 μm, respectively after release of the sensors (Fig. 5f). Thus, the total sinusoidal length of a single buckle is ~46.09 μm and the increment in length per buckle can be estimated to be ~11.09 μm after complete stretching. For a substrate of thickness *t*_s_ wrapped around any curved surface of radius *R*, the circumferential length covered by the bottom and top surfaces of the substrate when completely wrapped around is given by *L* and *L′*, respectively as


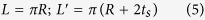


and schematically represented in Fig. 5d. The increment in circumferential length (Δ*L*) for a 100 μm thin film would approximately be then


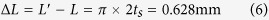


Thus, the buckles need to incorporate cumulatively a linear increment of ~628 μm for successful wrapping around the any curved surface for a 100 μm thick sensor. Hence, for a total increment of 628 μm, the estimated total no of buckles (*N*_b_) is ~60 resulting in a total buckle wavelength of *λ*_b_ × *N*_b_ = 2100 μm which in turn would be the total length (*L′*) required for a 100 μm thick film to cover a surface of radius *R*. Therefore, the minimum tensile bending radius, *R* can be estimated to be ~230 μm.

## Additional Information

**How to cite this article**: Maji, D. *et al.* Buckling assisted and lithographically micropatterned fully flexible sensors for conformal integration applications. *Sci. Rep.* **5**, 17776; doi: 10.1038/srep17776 (2015).

## Supplementary Material

Supplementary Information

Supplementary Video
