# Supplementary material for: Buckling assisted and lithographically micropatterned fully flexible sensors for conformal integration applications
Source: Sci Rep. 2015 Dec 7;5:17776. doi: 10.1038/srep17776 (PMC4671021; doi:10.1038/srep17776)
Supplement: Supplementary Information [file srep17776-s1.doc]

**Buckling assisted and lithographically micropatterned fully flexible sensors for conformal integration applications**

Debashis Maji^1^, Debanjan Das^2^, Jyoti Wala^1^, and Soumen Das^1,*^

^1^*School of Medical Science and Technology,*

^2^*Department of Electrical Engineering,*

*Indian Institute of Technology Kharagpur - 721302, India*

^*^*email:* *sou@smst.iitkgp.ernet.in*

**Supplementary File**

**Supplementary Data 1:**

**Thin film buckling on PDMS surface:** The built in compressive stress or thermal stress, *σ*_0_ developed over a film deposited over flat planar PDMS when cooled to room temperature *T* below the deposition temperature *T*_d_ is given by^14^

(S1)

where *α*_s_ and *α*_f_ are the coefficient of thermal expansion of substrate and film respectively and *E*_f_ and *ν*_f_ are the Young’s modulus and Poisson’s ratio of the deposited film. Formation of random sinusoidal buckles takes place when the compressive stress exceeds a certain critical stress value *σ*_crit_ during the cooling process after sputtering as given by^14^

(S2)

where, *E*_s_ and *v*_s_ are the Young’s modulus and Poisson’s ratio for PDMS substrate respectively.

Considering a deposition temperature (*T*_d_) of about 180 °C (as experimentally observed), the thermal stress (*σ*_0_) is about 14 GPa as estimated from equation (S1) when cooled to a room temperature of *T*= 25 °C and considering the other parameters as *E*_f_ = 213 GPa, *E*_s_ = 269 MPa(at the surface of PPDMS)^37^, *ν*_f_ =0.3, *α*_f_ =15×10^-6^ °C^-1^ and *α*_s_=310×10^-6^ °C^-1^ as obtained from standard literature. The critical stress (*σ*_crit_) required for the onset of the sinusoidal buckles can be estimated to be about 1.59 GPa from equation (S2).

**Supplementary Data 2:**

**Measurement of stress profile across a ridge surface:** For a non-planar ridge topology, the stress distribution and prediction of buckle patterns in either direction can be obtained from the analysis of array of long ridges parallel to the y-axis having a ridge of width 2*d* (Fig. 2e) and is given by^14^

$\sigma_{x}=-\sigma_{0}\left[1-\frac{\cosh(x/l)}{\cosh(d/l)}\right]$ (S3)

$\sigma_{y}=-\sigma_{0}\left[1-\nu_{f}\frac{\cosh(x/l)}{\cosh(d/l)}\right]$ (S4)

where *σ*_x_ and *σ*_y_ are the stress components in the *x* and the *y*-direction respectively and *x* is measured from the centre of the ridge and *l* is the transition length or buckle free length near the vicinity of the ridge edges having considerably low or no stress due to stress discontinuity resulting in no buckle formation. Fig. 2e shows a typical plot of the stress distribution in *x* and *y* directions as obtained through MATLAB® over a 700 µm ridge width (2*d*= 700 µm) and transition length *l* of 250 µm as observed experimentally.

In case of a pre-stretched film, the build in stress over the pre-stretched film due to thermal expansion effect is similar to that over un-stretched film. However, gradual release of prestrain along the ridge length (i.e. y-direction) induces additional stress component (*σ*_pre_) that modifies the total compressive stress only along the y-direction as given by

$\sigma_{0}'=\sigma_{0}+\sigma_{\text{pre}}=\sigma_{0}+(\varepsilon_{\text{pre}}\times E_{\text{f}})$ (S5)

where *ε*_pre_ is the externally applied prestrain. Assuming negligible shrinkage across the ridge width due to Poisson’s effect, the stress in the x-direction is expected to remain same. Using a similar analysis as before and considering an array of long ridges having a ridge width 2*d* and ridge length parallel to the direction of pre-stretching i.e. along y-axis, the stress components along *x* and *y* directions upon release of the initial prestrain may be evaluated as follows

$\sigma_{\text{x,pre}}=-\sigma_{0}\left[1-\frac{\cosh(x/l)}{\cosh(d/l)}\right]$ (S6)

$\sigma_{\text{y,pre}}=-\sigma_{0}'\left[1-\nu_{\text{f}}\frac{\cosh(x/l)}{\cosh(d/l)}\right]$ (S7)

where *x* is measured from the centre of the ridge and *l* is the transition length. Unlike previous case for planar surface transition length does not appear to exist after the release of pre-stretched film due to large compressive stress *σ*_pre_. Fig. 2j shows the MATLAB® simulated stress profile *σ*_x,pre_ and *σ*_y,pre_ over similar ridge structure after complete release of the initial prestrain.

Considering similar deposition conditions as earlier, the thermal stress (*σ*_0_) and critical stress (*σ*_crit_) may be estimated to remain the same i.e. ~ 14 GPa and ~1.59 GPa respectively whereas the total compressive stress (*σ*_0_') becomes as high as 90 GPa for a 35% prestrain as estimated from equation (S5) resulting in a significant change in *σ*_y,pre_.

**Supplementary Fig. S1:**


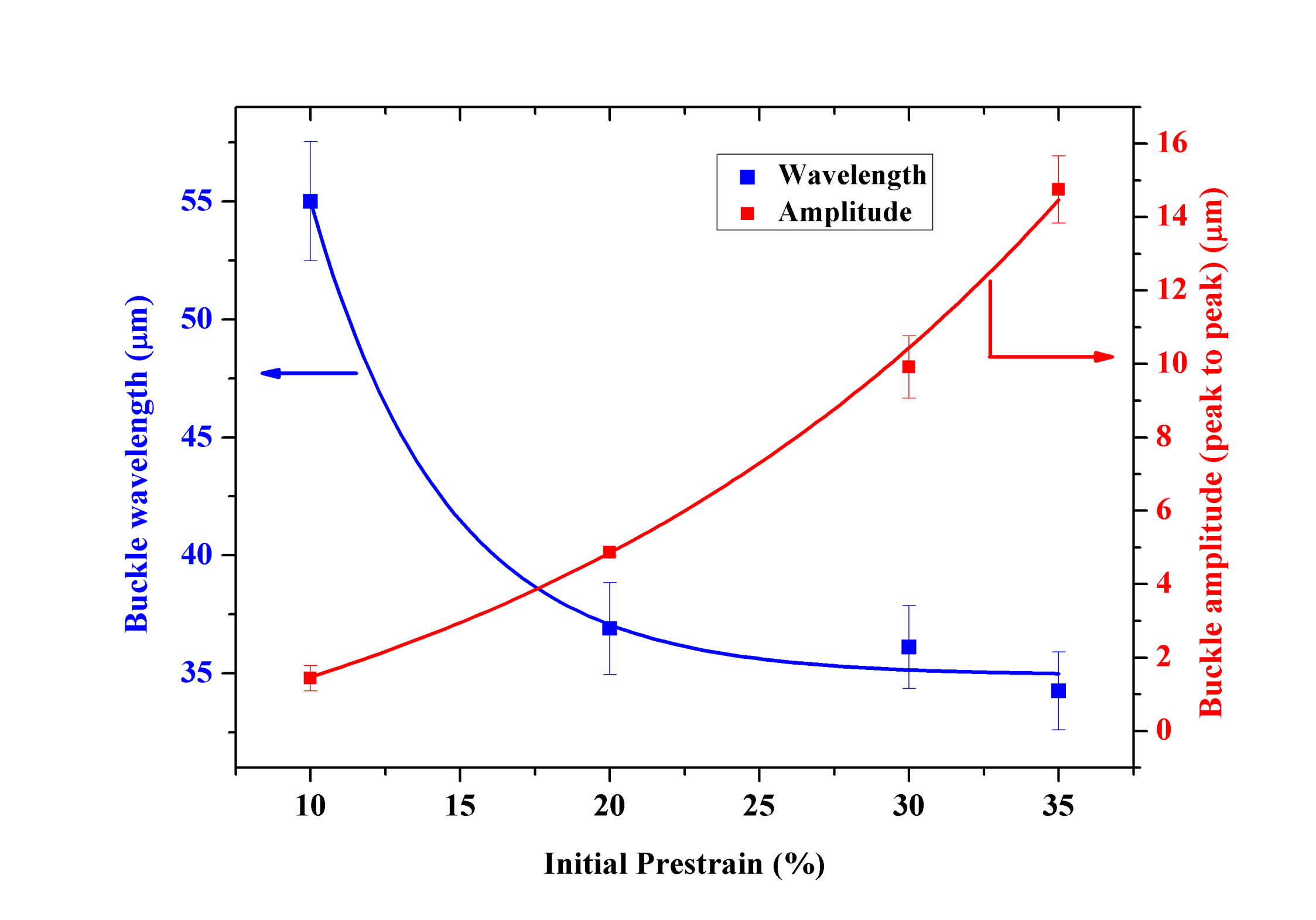


**Figure S1| Variation of buckle wavelength and amplitude.** Variation of wavelength and amplitude after complete release of prestrain for different initial applied prestrain. Low initial applied prestrain results in larger wavelength with smaller amplitude and vice-versa.

**Supplementary Fig. S2:**


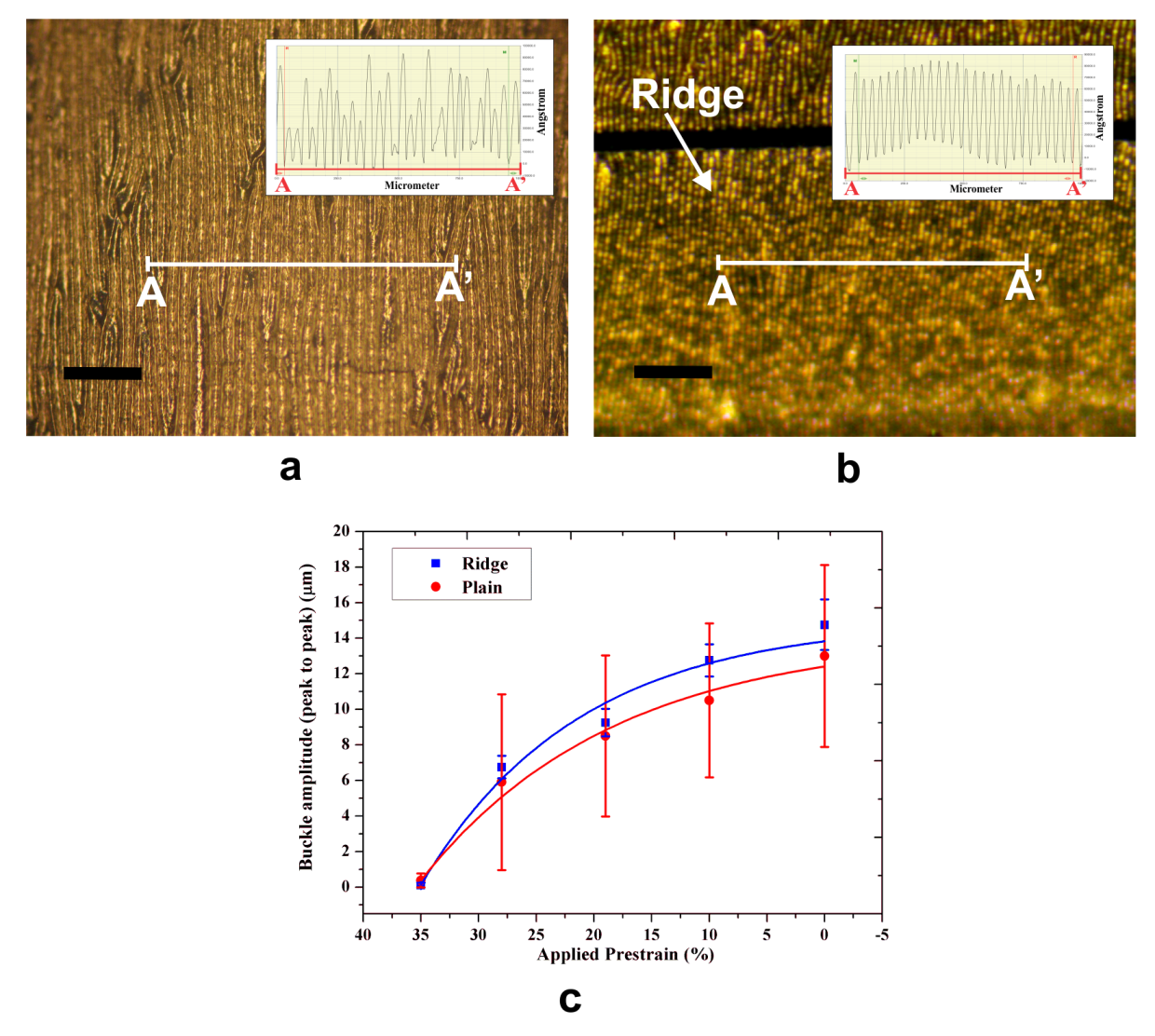


**Figure S2| Buckles formation over pre-stretched planar and ridge PDMS**. Microphotographs of buckles developed over (**a**) planar, (**b**) ridge PDMS surface after release from an initial prestrain of 35%. Inset graphs shows variation in surface profile of the two surfaces. (**c**) Variation of buckle amplitude with release of prestrain over planar and ridge topology. Scale bar, 200 µm (**a**,**b**).

**Supplementary Fig. S3:**


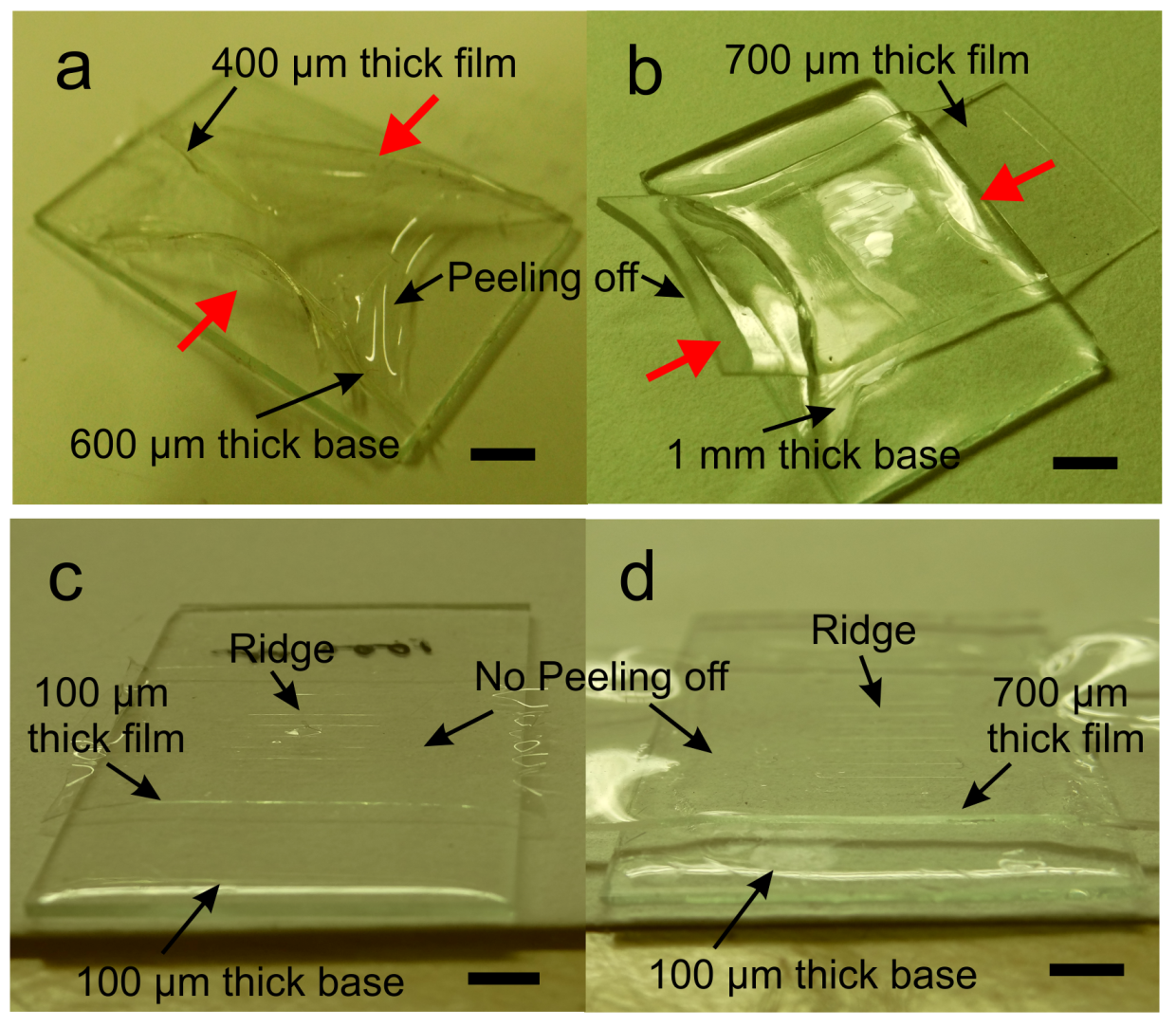


**Figure S3| Effect of base PDMS thickness on stability of pre-stretched films.** Pre-stretched films of (**a**) 400 µm and (**b**) 700 µm over 600 µm and 1mm base PDMS layer respectively gradually peeled off from the glass substrate along with the entire assembly due to high compressive stress of the pre-stretched film/base assembly. Stable films were obtained for all samples ranging from (**c**) 100 µm to (**d**) 700 µm thicknesses after fixing the pre-stretched films over 100 µm thin base PDMS layer which was plasma bonded to the glass substrate. Scale bar, 5 mm (**a**-**d**).

**Supplementary Fig. S4:**


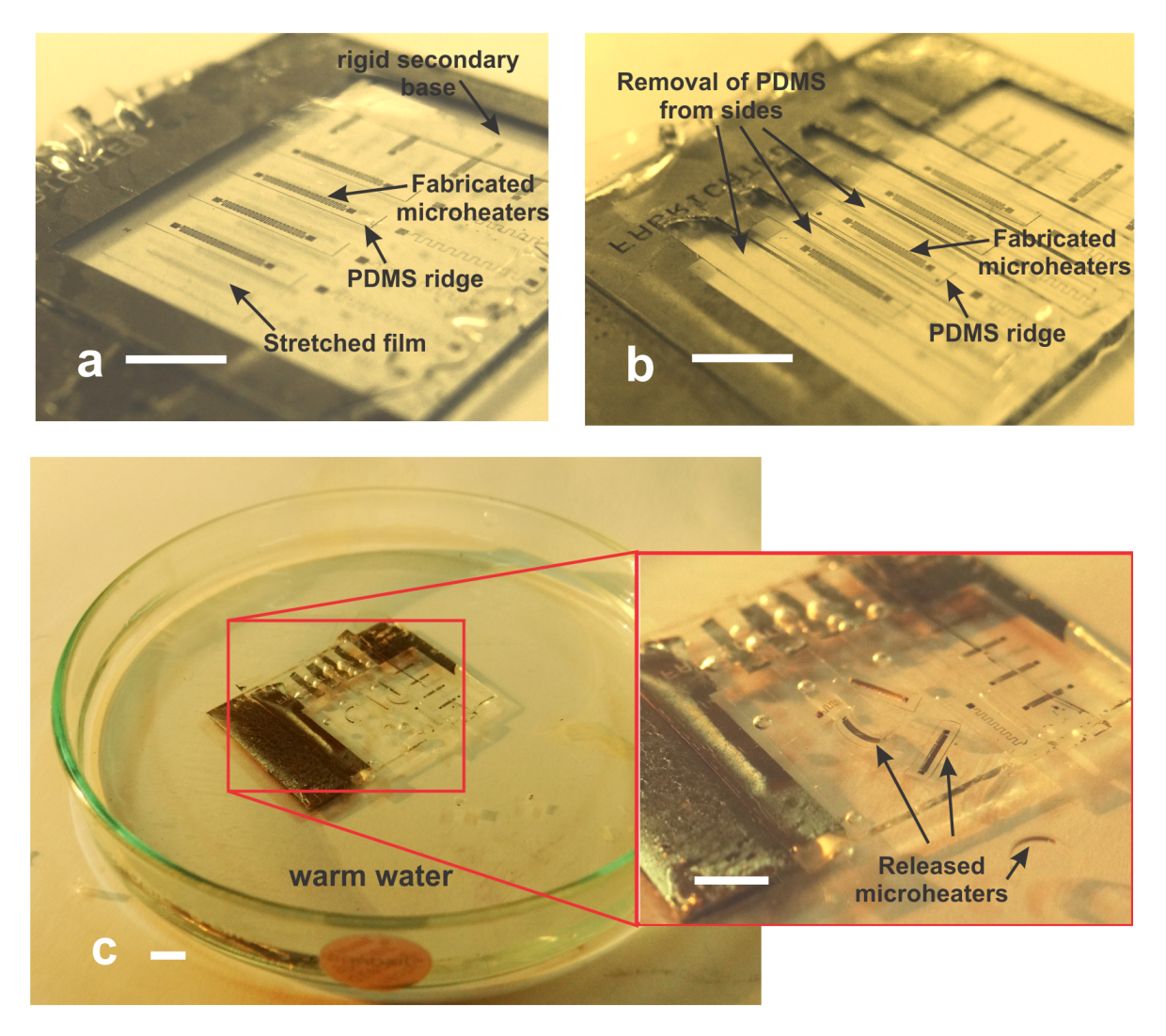


**Figure S4| Release of individual sensors.** Sequence of photographs illustrating (a) microheaters patterned over pre-stretched PDMS ridge film, (b) removal of PDMS between two ridges to facilitate easy seepage of water for sensor release and (c) dipping of entire assembly into warm water for stress free release of sensor through dissolution of PVA sacrificial layer. Inset shows magnified image of released sensors. Scale bar, 5 mm (**a,b,** magnified image in **c**), 2 mm (**c**).

**Supplementary Fig. S5:**


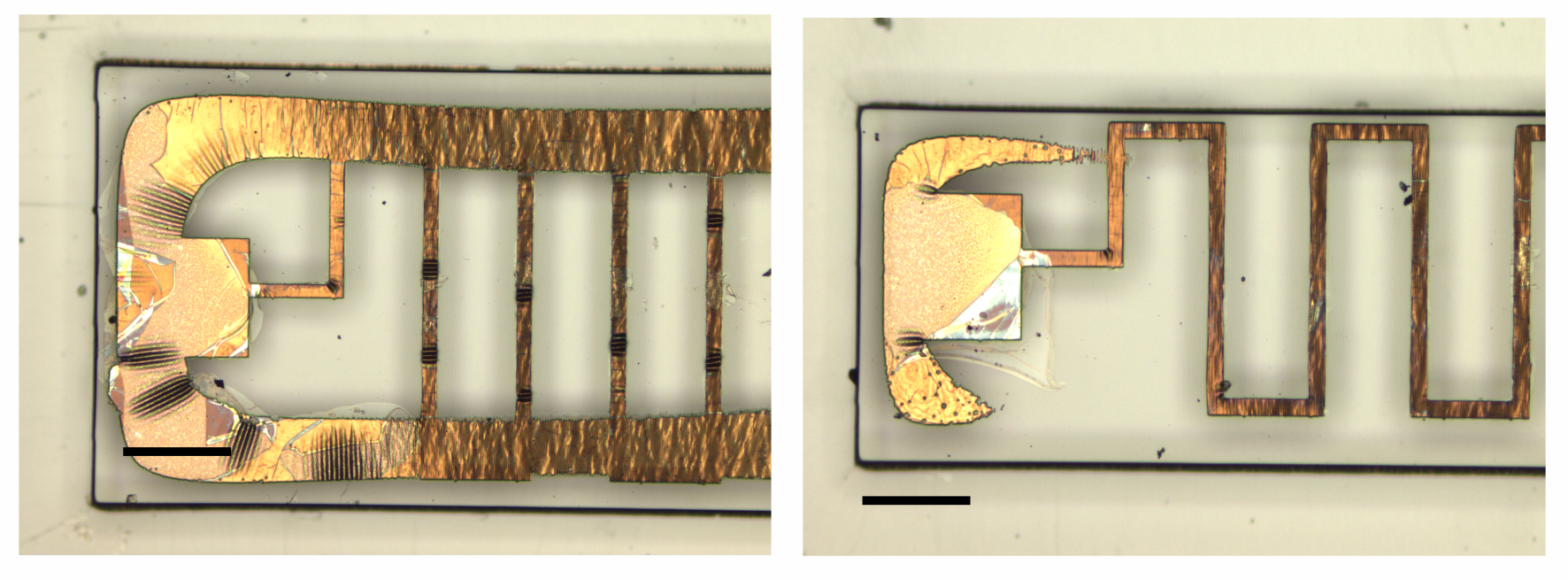


**Figure S5| Photolithography over thicker ridges.** Microphotographs of patterned structures over ridges of ~100 µm height showing the presence of unwanted metal thin film at the edges of the ridge due to accumulation of photoresist after spin coating. Scale bar, 400 µm.

**Supplementary Fig. S6:**


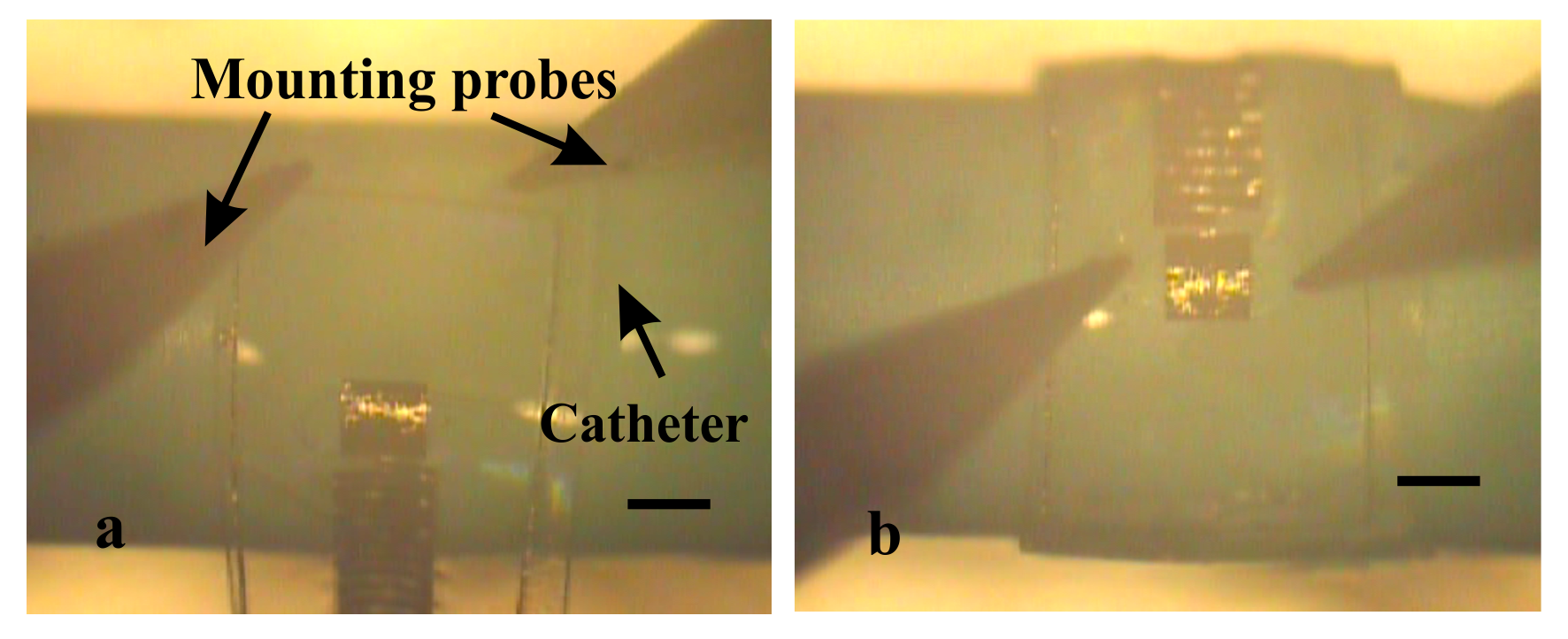


**Figure S6| Sensor Integration over catheter.** Microphotograph of sensor integration around the catheter tip showing (a) initial placing of the straight sensor at one end and (b) final wrapping of the sensor at the other end. Scale bar, 400 µm (**a,b**).
